# Effect of Matrix Metalloproteinase Inhibitors on the Dentin Bond Strength and Durability of a Two-Step Universal Adhesive System

**DOI:** 10.4317/jced.62406

**Published:** 2025-03-01

**Authors:** Mohammad Esmaeel Ebrahimi-Chaharom, Aida Moeinian, Mehdi Abed-Kahnamouei, Mehdi Daneshpooy, Mahmoud Bahari

**Affiliations:** 1 Professor, Department of Operative Dentistry, Faculty of Dentistry, Tabriz University of Medical Sciences, Tabriz, Iran; 2 Post-graduate Student, Department of Operative Dentistry, Faculty of Dentistry, Tabriz University of Medical Sciences, Tabriz, Iran; 3 Assistant Professor, Department of Operative Dentistry, Faculty of Dentistry, Tabriz University of Medical Sciences, Tabriz, Iran; 4 Dental and Periodontal Research Center, Tabriz University of Medical Sciences, Tabriz, Iran; 5 Department of Operative Dentistry, Faculty of Dentistry, Tabriz University of Medical Sciences, Tabriz, Iran

## Abstract

**Background:**

The aim of this study was to investigate the effects of using two types of matrix metalloproteinase inhibitors (MMPI) on the dentin bond strength and durability of a two-step universal adhesive (G2-BOND Universal Adhesive).

**Material and Methods:**

This study was conducted on 24 extracted molars, resulting in 144 samples. The occlusal surface of the teeth was cut perpendicularly to the longitudinal axis to expose the dentin. The samples were divided into 6 groups: Group 1 (Control, etch-and-rinse (ER)): bonding in the ER mode without MMPI; Group 2: bonding in the ER mode with chlorhexidine (CHX); Group 3: bonding in the ER mode with benzalkonium chloride (BAC); Group 4 (Control, Self-etch (SE)): bonding in the SE mode without MMPI; Group 5: bonding in the SE mode with CHX; Group 6: bonding in the SE mode with BAC. The entire dentin surface was restored with composite resin. Each group was further divided into two subgroups and either thermocycled for 500 or 10,000 cycles. The samples were cut into cylinders with a one square millimeter cross-sectional area and tested for microtensile bond strength (µTBS). Data was analyzed using 3-Way ANOVA and Games-Howell tests (*p*< 0.05).

**Results:**

There was a statistically significant difference in the mean µTBS based on the type of MMPI, aging method, and etching strategy. The mean µTBS in the Control group was significantly lower than in the CHX and BAC groups (*P*< 0.05). The mean µTBS was higher at 24 hours and in the ER group (*P*< 0.001). The effect of aging was consistent and decreasing (*P*> 0.05).

**Conclusions:**

The µTBS to dentin decreases after aging. However, the use of MMPI preserves bond strength to some extent in comparison to control groups after aging.

** Key words:**Universal adhesive, Matrix metalloproteinase, Bond strength, Chlorhexidine, Benzalkonium chloride.

## Introduction

Since the introduction of universal adhesives, they have gained increasing attention due to their ease of use and ability to bond to various substrates. However, the combination of essential components with different chemical properties in a single bottle leads to reduced stability, water sorption, phase separation, increased nanoleakage, and unsTable bond strength. Universal adhesives act as hydrophilic and permeable components which causes water to diffuse through the dentin even after polymerization (1).

Recently, a two-step universal adhesive namely G2-BOND Universal has been introduced with the aim of improving the strength and longevity of the substrate-adhesive interface. This system sets a new standard in all etching modes and offers comprehensive functionality from a unique combination of functional monomers devoid of HEMA for bonding to various substrates. It is also claimed that due to the two-step strategy and the presence of UDMA, it provides a more hydrophobic bonding layer that reduces water absorption and the risk of degradation (2).

The ability of dentin adhesives to bond to dentin depends on the formation of a hybrid layer composed of collagen fibers infiltrated with resin. Long-term studies have shown a decrease in bond strength over time, with the degradation of the hybrid layer cited as the main reason (3). Apart from external factors such as water absorption or oral fluid diffusion and polymer swelling, some endogenous proteolytic enzymes like matrix metalloproteinases (MMPs) are also responsible for the degradation of the hybrid layer. The stability of the bond decreases following the degradation of collagen fibers by MMPs (4). Therefore, inhibiting MMPs could be a key factor in solving this problem. MMPs are a family of 23 endogenous proteolytic enzymes whose activity is calcium and zinc dependent. The MMPs present in dentin are inactive and can be activated by changes in pH during the progression of carious lesions or adhesive protocols (5). Additionally, lower degrees of resin monomer penetration in dentin treated with the etch-and-rinse (ER) strategy lead to incomplete penetration of resin monomers and exposed collagen fibers at the bottom of the hybrid layer (6). Dentin MMPs can degrade these unprotected collagen fibers, also.

Form clinical perspective, MMP inhibitors such as chlorhexidine (CHX) and benzalkonium chloride (BAC) can play a significant role in the longevity of resin bonds to dentin (7,8). CHX sequesters cations like calcium and zinc, which are required for the activation of MMPs, and prevents the activity of MMPs in the dentin matrix (9). Studies have shown that the application of CHX has a broad-spectrum inhibitory effect on MMPs and significantly preserves the integrity of the hybrid layer formed by adhesives (10-12). However, several studies have reported a lack of effect or even a negative effect of CHX on the bond strength of adhesive systems (13-15).

BAC is a quaternary ammonium compound with antibacterial properties that has been introduced as an MMP inhibitor. Studies have shown that 0.5% and 1% BAC can inhibit MMPs in the hybrid layer and create a homogeneous adhesive layer without degeneration (8,16). The positive groups of BAC bind to the negatively charged functional groups of hydroxyapatites, collagen, and MMPs, leading to enzyme inactivation. Nevertheless, it has been reported that CHX carries a double positive charge while BAC carries a single positive charge, which may favor CHX (17). Despite this, Yaghmoor *et al*. (18), showed that adding MMP inhibitors (CHX and BAC) to adhesives improves the bond strength of resin to dentin. It appears that 1% BAC has a similar effect to CHX on bond strength.

Since information regarding the bond strength of two-step universal adhesives after aging conditions is limited, the aim of this study was to determine the microtensile bond strength (µTBS) and durability of composite resin to dentin using the two-step universal adhesive system (G2-BOND Universal) when using CHX and BAC as MMP inhibitors (MMPI).

## Material and Methods

This laboratory study was conducted on 24 human molar teeth, which were extracted due to impaction and periodontal disease. To control the impact of individual conditions on the samples, patients aged 20-35 years were selected. The teeth were visually examined and probed, and only healthy teeth without decay, fractures, or cracks were included in the study.

The teeth were cleaned with a brush and pumice and stored in a 0.5% chloramine T solution (Merck, Darmstadt, Germany) until the tests were performed. The teeth were sectioned under water perpendicular to the longitudinal axis to remove the occlusal enamel. To standardize the cut surfaces, they were polished with SiC paper (grit 600) under running water for 30 seconds.

The samples were randomly divided into 6 groups based on the MMP inhibitor application, and the etching strategy:

Group 1 (Control, ER): G2-BOND Universal adhesive (G2B) (GC, Tokyo, Japan) in ER mode without MMPI: After etching the dentin with 35% phosphoric acid (3M ESPE, St. Paul, MN, USA) for 15 seconds, and rinsing for 30 seconds, the dentin surface was dried using the blot drying method until visible moisture was removed.

Group 2: G2B Adhesive in ER mode with CHX: After etching the dentin similar to Group 1, 2% CHX (Consepsis, Ultradent, USA) was applied actively for 60 seconds using a microbrush on the etched dentin. The surface was then dried using the blot drying method until moisture was absorbed by capillarity.

Group 3: G2B Adhesive in ER mode with BAC: After etching the dentin similar to Group 1, BAC 1% (Merck, Darmstadt, Germany) was applied actively for 60 seconds using a microbrush on the etched dentin. The surface was then dried using the blot drying method until moisture was absorbed by capillarity.

Group 4: G2B Adhesive in self-etch (SE) mode without MMPI: G2B Adhesive was applied according to the manufacturer’s instructions using the SE method.

Group 5: G2B Adhesive in SE mode with CHX: All steps were similar to Group 2, except that bonding was applied using the SE method.

Group 6: G2B Adhesive in SE mode with BAC: All steps were similar to Group 3, except that bonding was applied using the SE method.

After applying the bonding according to the manufacturer’s instructions, the entire surface of the dentin was restored with Valux Plus composite (3M ESPE, St. Paul, MN, USA) in shade A1 to a height of 6 mm (in three layers, each approximately 2 mm thick). The thickness of each layer was measured with a probe. Each layer was cured separately for 40 seconds using the Demetron A2 light cure device (Kerr, Scafati, Italy).

In the next step, samples from each group were divided into two subgroups based on the aging method:

Subgroup 1: Samples were kept in distilled water at 37°C for 24 hours and then thermocycled between 5 and 55 °C for 500 cycles with a 10-second interval.

Subgroup 2: To simulate one year of aging, samples were thermocycled between 5 and 55 °C for 10,000 cycles with a 10-second interval.

Then, the samples were sectioned into cylindrical pieces with a cross-sectional area of 1 square millimeter and cut vertically.

The µTBS was measured using a micro-tensile testing machine (Bisco, Schaumburg, IL, USA) at a loading rate of 0.5 mm/min. After conducting the micro-tensile test, the failure pattern of the samples was examined under a stereo microscope (Nikon SMZ800, Tokyo, Japan) at 10x magnification: Type I: Cohesive failure in dentin, Type II: Cohesive failure in the composite, Type III: Adhesive failure, Type IV: Mixed failure.

-Statistical analysis 

SPSS version 20 was used for data analysis. First, the normality of the data was assessed using the Kolmogorov-Smirnov test. To compare the mean bond strength, three-way ANOVA and post-hoc tests (Bonferroni and Games-Howell) were used. A significance level of less than 5% was considered for all tests (*p* < 0.05).

## Results

Data related to µTBS in the experimental groups and subgroups are summarized in  Table 1. The Kolmogorov-Smirnov test for normality indicated that the distribution of µTBS was normal (*p* > 0.05).

The results of the three-way ANOVA showed that the effects of all three variables—aging (*p* < 0.001), etching strategy (*p* < 0.001), and type of MMP inhibitor (*p* = 0.002) on dentin bond strength were significant. The interaction effects of the above variables were not significant in any case (*p*> 0.05).

**Figure 1:**
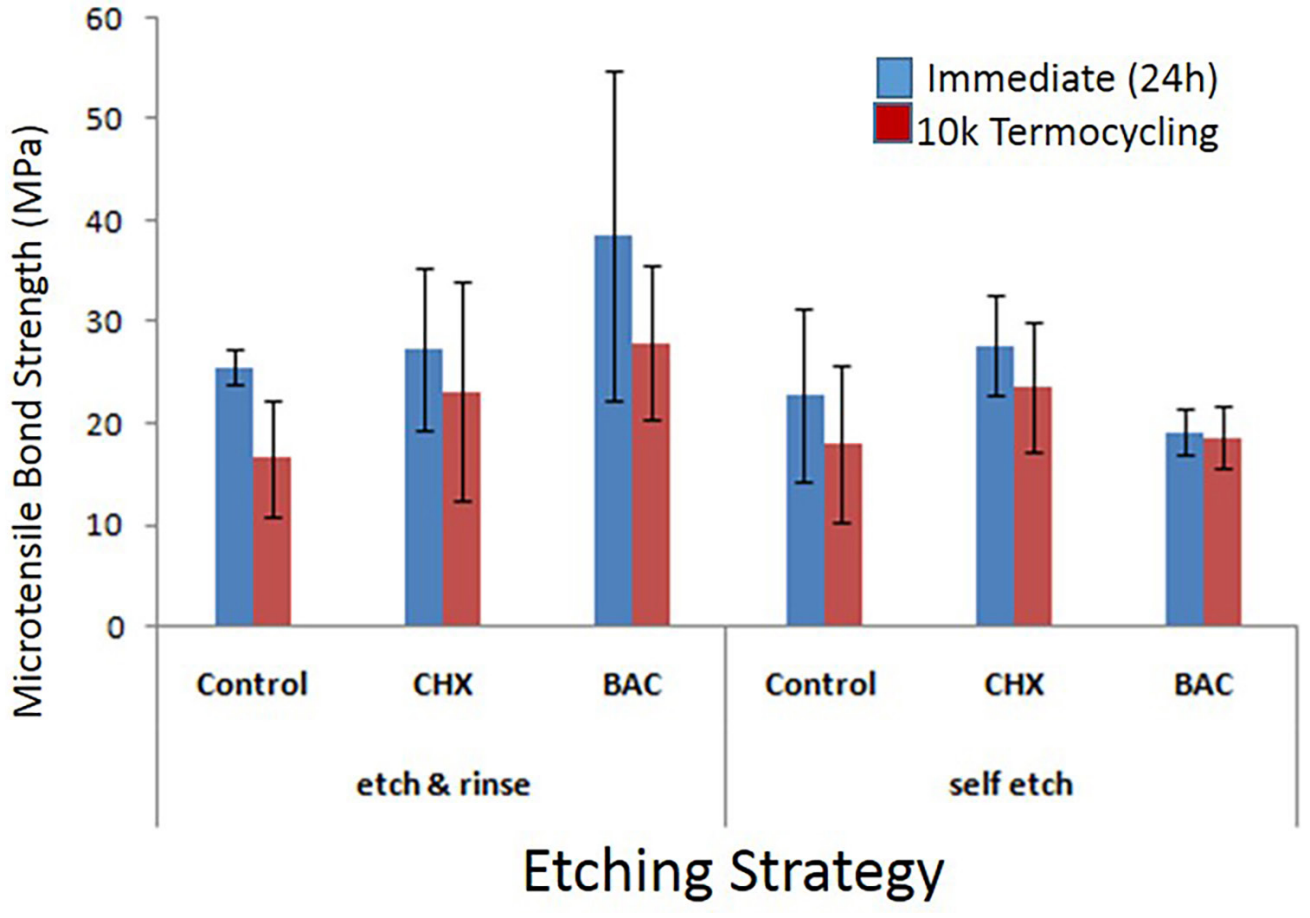
Error-bar graph of the Games-Howell test.

The results of the Games-Howell test indicated that there was a statistically significant difference between the mean dentin bond strength in the Control group and the mean in both the CHX and BAC groups, with the mean bond strength in the Control group being lower than that in the mentioned groups (*p* < 0.05). However, there was no statistically significant difference between the mean dentin bond strength in the CHX and BAC groups (Fig. 1).

**Figure 2:**
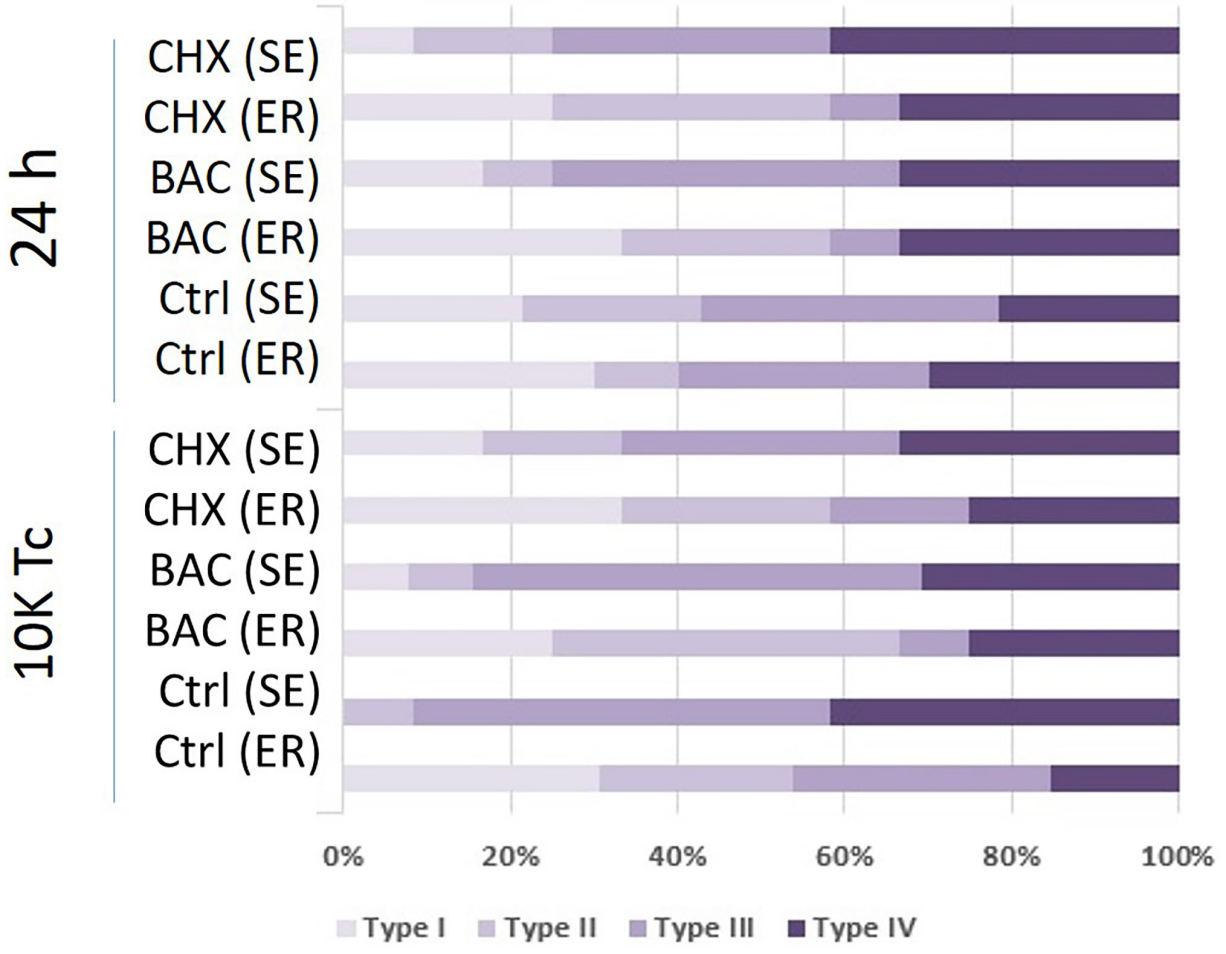
Distribution of failure patterns in experimental groups.

The most common failure pattern in the CHX group was mixed failure, while in the BAC group under SE conditions, the adhesive failure was predominant, and under ER conditions, the mixed pattern was observed. In the Control group, the most common failure pattern was adhesive (Fig. 2).

## Discussion

MMPs are zinc and calcium-dependent endopeptidases that enter the mineralized dentin matrix during tooth development. The release of these enzymes during bonding stages leads to the hydrolysis of extracellular matrix components. MMPs also lyse and remove collagen fibrils present in the hybrid layer (4). Therefore, it seems that the use of MMP inhibitors such as CHX and BAC on dentin can preserve the integrity of the collagen in the hybrid layer and play an important role in the longevity of resin bonds to dentin.

The present study showed that the use of CHX and BAC as MMP inhibitors results in greater bond strength compared to the Control groups. Previous studies have reported conflicting results regarding the effect of CHX on the bond strength of adhesives. Some studies have reported that CHX increases bond strength (19,20), while others have shown the opposite (21,22). Zheng *et al*., in a study on the effect of three types of exogenous MMP inhibitorss on the µTBS of 5th generation adhesives, demonstrated that CHX compared to other MMPIs (doxycycline 2% and proanthocyanidin 5%) significantly increased bond strength at 24-hour and 3-month intervals. They showed through immunolabeling and FESEM observations, the densest hybrid layer, the highest amount of exposed collagen and the greatest penetration of monomer into the interstitial space of collagen fibers in the CHX group, which may explain the highest bond strength in this group compared to other MMPIs (19). Additionally, Fasya *et al*., showed that using 2% CHX for 30 seconds before applying universal bonding using the ER technique increased bond strength. Furthermore, increasing the application time of CHX to 60 seconds resulted in further improvement in bond strength values, and beyond this time, there was no significant effect. Therefore, the ideal application time for CHX on dentin is 60 seconds, which was also applied in the present study (23). However, contrary to the results of the present study, Lenzi *et al*., found that the use of 2% CHX for 60 seconds had no effect on the immediate bond strength of Adper Single Bond adhesive in permanent and primary teeth with carious and healthy dentin (24). Ebrahimi *et al*. (25), also showed that the use of CHX had no effect on the bond strength of ER and SE adhesives.

Similar to CHX, studies have reported conflicting results regarding the effect of BAC. Tekçe *et al*., reported that adding BAC %1 to etching acid had no effect on the bond strength of mild and ultramild universal adhesives to dentin (16). Comba *et al*. (26), demonstrated that all formulations from the All Bond Universal adhesive containing BAC reduced the expression of matrix MMPs. However, a general trend of increased enzymatic activity after aging was clearly observed. Therefore, the µTBS test, despite the increase in immediate bond strength, showed a reduction in bond strength over time in all tested groups. The study by Sabatini *et al*., also indicated the inhibition of proteolytic activity in dentin and improved tensile bond strength following the use of BAC at various concentrations when added to the compositions of Optibond Solo Plus and All-Bond 3 (27). However, adding a new component to the adhesive composition can lead to a decrease in the degree of conversion of the monomer and reduced mechanical properties. Therefore, in the present study, BAC was used as a rinse before applying the adhesive. In line with the current study, Sabatini *et al*. (8) in another study showed that in the ER method, the use of BAC at concentrations of 0.5% and 1% for 60 seconds could inhibit MMP activity by 31% and 54%, respectively, thereby preserving the adhesive bond strength over a one-year period.

Tekçe *et al*., demonstrated that the type of bonding is important in determining the effect of MMP inhibitors. In their study, under the ER condition, the use of BAC and CHX resulted in the highest bond strength of Single Bond Universal adhesive at 24 hours. In the All Bond universal adhesive, BAC also significantly increased bond strength in the ER method. In both bonding methods, bond strength values in the ER method were higher than those in the SE method (28). This was also confirmed in the present study, as the bond strength in the ER groups was greater than in the SE groups.

Another noTable finding in the present study was that aging significantly reduced bond strength, regardless of the type of MMP and etching strategy used. Consistent with the current study, Tekçe *et al*., also reported a decrease in bond strength over one year across all groups (28). Bedir *et al*. (29), showed that after six months, none of the MMP inhibitors caused a change in µTBS compared to the control group, regardless of the type of universal bonding and etching strategy employed. Komori *et al*. (30), by examining the effect of using 2% CHX for 60 seconds on the bond strength of Scotchbond Multi-Purpose-MP and Single Bond 2-SB, demonstrated that CHX does not increase bond strength immediately after bonding. However, after six months, CHX reduces the loss of bond strength.

## Conclusions

It can be concluded that:

1. The use of CHX and BAC as MMP inhibitors increases the immediate µTBS of G2B compared to the control group.

2. The use of CHX and BAC as MMP inhibitors reduces the loss of µTBS after aging.

3. µTBS decreases due to aging.

4. The ER method for using G2B universal adhesive results in greater bond strength compared to the SE method.

## Figures and Tables

**Table 1:** Mean ± standard deviation of microtensile bond strength of experimental groups.

Adhesive mode	MMP inhibitors	Bond strength (MPa)	
		24 hours	10k TC
etch & rinse	Control	25.73±1.72	16.68±5.72
	CHX	27.43±8.08	23.30±10.71
	BAC	38.65±16.20	28.06±7.71
self-etch	Control	22.87±8.65	18.03±7.78
	CHX	27.71±4.92	23.67±6.42
	BAC	19.29±2.34	

## Data Availability

The datasets used and/or analyzed during the current study are available from the corresponding author.
